# Exploring Clinical Decision-Making Competencies of Emergency Nurses in Trauma Care in Indonesia: Qualitative Study

**DOI:** 10.2196/74282

**Published:** 2025-06-25

**Authors:** Saharuddin Saharuddin, Elly Nurachmah, Masfuri Masfuri, Dewi Gayatri, Amelia Kimin, Muhammad Sakti, Sanisah Binti Saidi, Sri Yona

**Affiliations:** 1 Faculty of Nursing, Universitas Indonesia, Gedung Pascasarjana FIK UI & Laboratorium, Jl. Prof. Dr. Bahder Djohan, Kampus UI Depok, Depok, 16424, Indonesia, 62 85399772768, 62 217864124; 2 Faculty of Medicine, Universitas Hasanuddin, Makassar, Indonesia; 3 Faculty of Nursing, International Islamic University Malaysia, Kuantan, Malaysia

**Keywords:** clinical decision-making, emergency nursing, trauma care, Indonesia, emergency nurse

## Abstract

**Background:**

Clinical decision-making is vital for emergency nurses, especially in trauma care that requires swift, accurate actions. In Indonesia, where resources are limited, little is known about how nurses manage such challenges.

**Objective:**

This study aimed to explore the clinical decision-making competencies of emergency nurses in trauma care, focusing on challenges, strategies, and influencing factors.

**Methods:**

This was a qualitative study using semi-structured interviews with 16 emergency nurses, complemented by observations and document analyses. Data were analyzed thematically, with triangulation, thereby ensuring validity.

**Results:**

Six key themes emerged: (1) recognize cues; (2) analyze cues; (3) prioritize hypothesis; (4) generate solutions; (5) take actions; and (6) evaluate outcomes. These highlight the adaptive and multidimensional nature of decision-making in emergencies.

**Conclusions:**

The decision-making of emergency nurses integrates analysis, prioritization, collaboration, and reflection. Enhanced training, such as simulation-based learning, and addressing systemic barriers can improve competencies. Policymakers should provide adequate resources and robust standards to support nurses under pressure.

## Introduction

Clinical decision-making is at the core of nursing practice, particularly in emergency departments (EDs), which demand speed and accuracy. In these settings, nurses must recognize clinical cues, prioritize actions, make rapid decisions, and evaluate outcomes to ensure patient safety and quality of care [1]. These decisions are often made in complex and uncertain situations where errors can have fatal consequences [2].

Numerous studies have highlighted the importance of experience, intuition, and situational awareness in nurses’ decision-making. Intuition is frequently used by experienced nurses through pattern recognition developed over years of clinical practice. In contrast, less experienced nurses tend to rely on more structured analytical approaches [2,3]. Additionally, clinical decision-making is influenced by organizational factors, workplace culture, education, and individual autonomy [4].

Although clinical decision-making has been extensively studied, most research has focused on developed countries. Research in Indonesia indicates that nurses in EDs face challenges such as resource limitations, high patient loads, and inadequate training [5]. Furthermore, clinical experience and the use of advanced medical technology are critical factors in enhancing decision-making capabilities in emergency situations [2]. However, there remains a knowledge gap in understanding how nurses in developing countries manage these challenges in daily practice.

This study aims to explore the clinical decision-making competencies of emergency nurses in trauma care in Indonesia, focusing on the challenges, strategies, and supporting factors that influence their clinical decisions. Using a qualitative approach, this research seeks to contribute new insights to the global understanding of nurses’ clinical decision-making in high-pressure environments

## Methods

### Study Design

This study employed a qualitative approach with an exploratory design to gain an in-depth understanding of the clinical decision-making competencies of ED nurses in trauma care. Data were collected through semi-structured in-depth interviews with 16 ED nurses with at least 2 years of work experience in provincial referral hospitals in Indonesia.

### Data Collection

#### In-Depth Interviews

Interviews were conducted in person using open-ended questions designed to explore nurses’ experiences in recognizing patient cues, prioritizing actions, and evaluating intervention outcomes. Each interview lasted 45‐60 minutes and was recorded for further analysis with the participants’ consent.

#### Observation

Observations were carried out in ED settings during active working hours to document how nurses recognized clinical signs, interacted with medical teams, and made clinical decisions in real-time situations.

#### Documentation

Relevant documents such as triage protocols, medical records, and operational policies were analyzed to support data from interviews and observations.

### Data Analysis

Data were thematically analyzed through verbatim transcription, coding, theme development, and triangulation of interviews, observations, and documents to ensure validity and capture nurses’ experiences influencing clinical decision-making in EDs.

### Ethical Considerations

Ethical approval was obtained from the University of Indonesia Ethics Committee (KET-036/UN2.F12.DI.2.1/PPM.00.02/2024), and all participants provided informed consent after understanding the study’s purpose, confidentiality, and their right to withdraw without consequences. The data were de-identified.

## Results

### Theme 1: Recognize Cues

#### Subtheme 1.1: Identifying Key Data

Nurses prioritized vital signs such as respiratory rate and blood pressure. One participant stated, "I usually start by looking at their breathing, whether it’s normal or not. If there are additional sounds, it needs immediate attention” (R2). Observations confirmed that prioritization of vital signs was supported by triage protocols.

#### Subtheme 1.2: Integrating Clinical Information

Nurses used data from medical histories and families. “If the patient is unconscious, we ask the family if there’s a history of conditions like heart disease,” explained a participant (R9). Patient documentation indicated that such data helped establish initial interventions.

### Theme 2: Analyze Cues

#### Subtheme 2.1: Correlating Symptoms with Clinical Conditions

Symptoms such as limb pain were analyzed as indications of fractures. “If there’s significant swelling and the patient can’t move, it’s usually a fracture,” noted one nurse (R6). This finding aligned with observed actions in cases of extremity trauma.

#### Subtheme 2.2: Projecting Risks of Complications

Nurses predicted complications such as internal bleeding. “Sometimes patients appear stable, but we need to consider possible bleeding,” remarked a participant (R13). Clinical documentation supported these evaluations.

### Theme 3: Prioritize Hypothesis

#### Subtheme 3.1: Justifying Priorities

Priority decisions were based on the critical condition of patients. “If the patient is short of breath, we prioritize them because they can quickly go into respiratory arrest,” said one respondent (R7). Observations showed that priorities were determined using triage algorithms.

#### Subtheme 3.2: Team Collaboration

Collaboration with doctors and medical teams was crucial for setting priorities. “We always discuss critical conditions with the doctors,” said a nurse (R4). Team meeting documentation demonstrated alignment in decision-making.

### Theme 4: Generate Solutions

#### Subtheme 4.1: Adapting Care Plans

Nurses modified plans based on changes in patients’ conditions. “We continuously evaluate. If there’s a change, we adjust,” said a respondent (R10). Observations noted immediate actions for adaptation.

#### Subtheme 4.2: Adhering to Nursing Standards

Interventions were carried out following standard operating procedures. “We always refer to protocols, for example, for IV insertion,” stated a nurse (R11). Protocols documented consistent application of standards.

### Theme 5: Take Actions

#### Subtheme 5.1: Dynamic Monitoring

Nurses regularly monitored changes in vital signs. “After the intervention, we recheck to see if there’s improvement,” said one participant (R5). Observations noted that evaluations were conducted every 15 minutes in critical cases.

#### Subtheme 5.2: Response-Based Modifications

If outcomes were unsatisfactory, plans were revised. “If oxygen saturation remains low despite oxygen therapy, we consider other interventions,” said a respondent (R20). Documentation showed the use of evaluation results to adjust actions.

### Theme 6: Evaluate Outcome

#### Subtheme 6.1: Real-Time Documentation

Nurses documented all actions immediately after implementation. “Whatever we do, it must be written immediately,” said one participant (R2). Electronic medical record systems supported this process.

#### Subtheme 6.2: Patient and Family Education

Education was provided to improve patients’ understanding. “We explain the procedures to reassure families,” noted a respondent (R7). Observations recorded frequent family involvement in care processes.

## Discussion

### Principal Findings and Comparison With Previous Works

This study identified 6 key themes underlying emergency nurses’ clinical decision-making competencies in the management of trauma patients. The findings provide a comprehensive picture of the decision-making process that is not only reactive, but also proactive and reflective, according to the complexity of the situation in the ED. This emphasizes that clinical decision-making is a multidimensional competency that requires the integration of knowledge, clinical experience, intuition, and communication and collaboration skills.

The first and second themes, recognizing clinical cues and analyzing signs and symptoms, emphasize the importance of keen observation and critical analysis under highly dynamic conditions. This is in line with the clinical decision-making model developed by Tanner [1], which emphasizes the process of pattern recognition and situational awareness as the main foundations of clinical decision-making [6]. In addition, this finding also reinforces Benner and Tanner’s [3] concept that underlines the role of experience in building clinical intuition that helps nurses make quick and appropriate decisions in critical circumstances.

In problem prioritization, nurses not only rely on objective data from vital signs and medical history, but also apply empirical frameworks such as triage systems and emergency protocols [4]. Interprofessional collaboration was the key to success in this process, in line with the literature that shows that effective communication between medical teams contributes significantly to the reduction of clinical errors and improved patient outcomes [7].

The implementation of the care plan and the evaluation of outcomes highlighted the need for flexibility in responding to real-time changes in the patient’s condition. This adaptive approach is encouraging a continuous decision-making process based on the monitoring and reflection of clinical data [8,9]. The ability to modify interventions based on patient response also reflects the professionalism and ethical responsibility of nurses who are not only protocol-oriented, but also in the real context of the patient [10].

Strong documentation and education support the effectiveness of the decision-making process [11]. Accurate documentation not only ensures the continuity of care but also serves as legal evidence and a means of communication between teams [12,13]. Patient and family education, despite time constraints in the ED, is instrumental in improving adherence and social support, both of which are important in the recovery phase [14,15]. This is in line with the principle of holistic care that integrates physical, psychological, and social aspects in patient care.

### Limitations

The focus on hospitals in Indonesia with specific local resource and cultural characteristics limits the broad generalizability of the findings. The limitations of qualitative data in the form of potential subjective bias of participants and researchers need to be anticipated by triangulation methods, and the results need to be validated more broadly in future studies.

Future research should employ mixed methods and expand samples across diverse health care settings. The SAHAR (steps to acquaintance of hypothesis in achieving recovery) model, derived from this study, should be refined and tested as a training intervention to enhance clinical decision-making, particularly in high-pressure environments like EDs.

### Conclusions

This study presents the clinical decision-making process of emergency nurses in trauma care and introduces the SAHAR model as a novel framework to enhance adaptive decision-making. The integration of this model into nursing education and clinical practice is recommended to improve decision-making competencies, supported by adequate resources and standardized procedures. Further research is needed to validate and refine the model across diverse health care settings.

## Abbreviations

ED: emergency department
SAHAR: steps to acquaintance of hypothesis in achieving recovery
